# Treatment and outcomes of POEMS syndrome: changes in the past 20 years

**DOI:** 10.1038/s41408-021-00540-1

**Published:** 2021-08-14

**Authors:** Yan-ying Yu, Xue-min Gao, Hao Zhao, Hao Cai, Jun Feng, Lu Zhang, Xin-xin Cao, Daobin Zhou, Jian Li

**Affiliations:** Department of Hematology, Peking Union Medical College Hospital, Chinese Academy of Medical Science & Peking Union Medical College, Beijing, China

**Keywords:** Myeloma, Therapeutics

**Dear Editor**,

Polyneuropathy, organomegaly, endocrinopathy, monoclonal gammopathy, and skin changes (POEMS) syndrome is a rare plasma cell dyscrasia with multiorgan involvement. In the past two decades, great progress has been achieved in the diagnosis, treatments, and outcomes of POEMS syndrome [1–3]. The introduction of the VEGF test has contributed to the accurate diagnosis of the disease and the timely assessment of treatment responses [4]. The melphalan plus dexamethasone regimen has been shown to have promising neurologic and organ responses [5], while high-dose melphalan followed by autologous stem cell transplantation (ASCT) makes it possible to achieve a deeper hematological response and longer survival [6]. In recent years, new agents, including immunomodulatory drugs (IMiDs) and protease inhibitors (PIs), have also been increasingly applied in patients with POEMS syndrome, achieving similar treatment responses compared with ASCT with reduced side effects [7].To describe the changes in a large-scale setting and outline the trends in disease management, we analyzed the changes in baseline characteristics, the first-line treatment options, and the outcomes of the patients who were newly diagnosed with POEMS syndrome in our institution in the past two decades.

A total of 621 patients newly diagnosed with POEMS syndrome between 1 January 2000 and 31 December 2019 at Peking Union Medical College Hospital were included in this study. Data were extracted from a prospectively maintained database consisting of patients who satisfied the POEMS syndrome diagnostic criteria described by Dispenzieri [8]. The last follow-up date was 1 December 2020. All patients signed a written informed consent form. The study was approved by the Institutional Review Board of Peking Union Medical College Hospital and conducted in accordance with the Declaration of Helsinki.

The study cohort was divided into three chronological cohorts that spanned similar time intervals based on the year of diagnosis: 2000–2009, 2010–2014, and 2015–2019, and there were 81, 221, and 319 patients included, respectively, which showed a significantly increasing trend. The baseline characteristics of the entire cohort (*n* = 621) and by diagnosis period are presented in Table 1. The median age at diagnosis in 2000–2010, 2010–2014, and 2015–2019 groups was 45, 48, and 51 years (*p* = 0.021). The time interval from symptom onset to diagnosis was 23.0 in the entire cohort and 15.9, 12.6, and 12.2 months (*p* = 0.037) in the three cohorts. In the three groups, 47 (58.0%), 86 (38.9%), and 65 (20.4%) patients used glucocorticoids before, mainly because of misdiagnosis.

**Table 1:** Baseline and treatment characteristics of the entire cohort and by time period.

Baseline characteristics	Entire cohort (*n* = 621)	Before 2010 (*n* = 81)	2010–2014 (*n* = 221)	2015–2019 (*n* = 319)	*P*, entire cohort
Age, y, median (range)	50 (21–83)	45 (38–83)	48 (26–81)	51 (21–78)	**0.021**
Male, %	61.2	59.6	64.9	59.0	0.351
**Symptom onset to diagnosis,*m*, median(range)**	12.5 (1–278)	15.9 (1–124)	12.6 (1–278)	12.2 (1–206)	**0.037**
**Polyneuropathy**					0.058
ONLS > 4, %(n)	34.1 (601)	38.0 (71)	39.3 (214)	29.7 (316)	
**Organomegaly**					
Hepatomegaly, %	41.0 (604)	52.5 (81)	47.8 (-220)	32.4 (303)	**<0.001**
Splenomegaly, %	65.3 (610)	73.0 (81)	69.4 (221)	59.9 (308)	**0.014**
Lymphadenopathy, %	62.9 (607)	73.0 (81)	61.8 (220)	60.5 (306)	0.064
**Castleman disease, % (*n*)**	64.8 (142)	63.2 (38)	55.8 (43)	72.1 (61)	0.222
**Monoclonal plasma cell disorder**					
IgA type heavy chain, %	61.3 (621)	61.6 (81)	61.8 (221)	60.9 (319)	0.972
λ light chain, %	96.5 (621)	99.0 (81)	96.9 (221)	95.3 (319)	0.160
SPE > 5 g/L, %	10.0 (621)	10.0 (81)	10.5 (221)	9.7(319)	0.967
BMPC > 10, %(n)	3.2 (532)	2.5 (80)	3.3 (215)	3.4 (237)	0.927
**Skin changes**					
Hemangioma, %	56.3 (596)	36.7 (80)	61.9 (219)	58.4 (297)	**<0.001**
Hyperpigmented, %	88.6 (602)	87.0 (80)	91.6 (219)	87.1 (303)	0.230
Hypertrichosis, %	63.0 (495)	60.0 (80)	53.8 (207)	73.6 (208)	**<0.001**
**Extravascular volume overload**					
Peripheral edema, %	84.1 (604)	87.6 (79)	89.5 (221)	78.9 (304)	**0.002**
Ascites, %	46.2 (571)	56.7 (79)	44.7 (219)	43.8 (273)	0.077
Pleural effusion, %	36.5 (563)	44.3 (79)	41.2 (218)	29.9 (266)	**0.007**
Papilledema, %	57.0 (401)	49.1 (74)	55.9 (190)	55.4 (137)	0.484
eGFR <30 mL/min/1.73 m^2^, %	5.1 (573)	3.7 (81)	5.0 (220)	5.5 (272)	0.807
Albumin ≤32 g/L,%	21.7 (521)	31.1 (67)	26.9 (212)	14.5 (242)	**<0.001**
Osteosclerosis, %	55.6 (486)	45.2 (54)	58.5 (163)	56.2 (269)	0.189
Pulmonary hypertension, %(n)	60.5 (377)	63.3 (60)	57.1 (156)	62.7 (161)	0.528
**VEGF**^**1**^	**(467)**	**(56)**	**(210)**	**(201)**	
Serum VEGF, pg/mL, median, range	4943 (50–21,316)	2730 (112–13,867)	4250 (92–21,316)	4964 (50–18,841)	**<0.001**
VEGF > 2000 pg/mL, %(n)	78.6	60.7	81.0	81.1	**0.002**
**Risk stratification,*n***	**(324)**	**(48)**	**(147)**	**(129)**	
Low risk, %	15.4	18.8	19.0	10.1	0.095
Medium risk, %	30.6	29.2	29.3	32.6	0.817
High risk, %	54.0	52.1	51.7	57.4	0.615
**GC treatment history, %**	31.9 (621)	58.0 (81)	38.9 (221)	20.4 (319)	**<0.001**
**Treatment,*n***	**(621)**	**(81)**	**(221)**	**(319)**	
ASCT	220	20	105	95	**<0.001**
No induction	145	13	74	58	**0.372**
IMiD induction	52	0	24	28	
PI induction	9	1	2	6	
Melphalan induction	14	6	5	3	
Melphalan	101	41	41	19	**<0.001**
MP	12	12	0	0	
MDex	89	29	41	19	
IMiDs	193	3	70	120	**<0.001**
LDex	182	3	65	114	
TDex	11	0	5	6	
PI	84	0	3	81	**<0.001**
BD	81	0	3	78	
Lxd	3	0	0	3	
Other^2^	23	17	2	4	**<0.001**

^1^The upper limit of the normal serum VEGF range was 600 pg/mL in our institute.

^2^ Other forms of therapy include radiation therapy, plasmacytoma resection, glucocorticoid only, CTX, etc.

*ONLS* Overall Neuropathy Limitations Scale, *SPE* serum protein electrophoresis, *VEGF* vascular endothelial growth factor, *IMiDs* immunomodulatory imide drugs, *PI* protease inhibitor, *GC* glucocorticoid, *MP* melphalan with prednisone, *MDex* melphalan with dexamethasone, *LDex* lenalidomide with dexamethasone, *TDex* thalidomide with dexamethasone, *BD* bortezomib with dexamethasone, *Lxd* ixazomib with dexamethasone, *ASCT* autologous stem cell transplantation.

Neuropathy was less severe in 2015–2019 than in the other periods, and 35.8, 41.7, and 29.6% of the patients in each group were evaluated as having Overall Neuropathy Limitations Scale (ONLS) > 4 (*p* = 0.001). The incidence of hepatomegaly and splenomegaly significantly decreased over time, as well as symptoms related to extravascular volume overload, such as peripheral edema and pleural effusion (*p* < 0.05). The proportion of albumin ≤32 g/L also decreased from 31.1 and 26.9–14.5% (*p* < 0.01). However, there was no significant difference in the incidence of renal dysfunction (5.1%), osteosclerosis (55.6%), or pulmonary hypertension (60.5%). The incidence of skin changes, such as angioma and hypertrichosis, increased, which may be attributed to the increased understanding of the disease and more careful physical evaluation. The median serum VEGF levels in the three cohorts were 2730, 4250, and 4964 pg/mL (*p* < 0.001). The risk stratification of the patients was performed according to the method described in the prognostic study by Wang et al. [9]. Risk factors included age > 50 years, pulmonary hypertension, pleural effusion (each 1 point), and estimated glomerular filtration rate <30 mL/min/1.73 m^2^ (2 points). A total score of 0 is considered low risk, 1 is considered medium risk, and 2–4 is considered high risk. Overall, 15.4, 30.6, and 54.0% of the patients were of low, medium, and high risk, with no significant constituent difference between any of the groups; the patients of high risk accounted for 52.1, 51.7, and 57.4% in each period (*p* = 0.615).

A total of 220 patients (35.4%) received high-dose melphalan followed by ASCT as first-line therapy, and the other 401 received non-ASCT regimens. The prevailing first-line regimen before 2010 was melphalan with glucocorticoids (50.6%, including MDex in 35.8% of the patients and MP in 14.8% of the patients), replaced by ASCT in the 2010–2014 period (47.5%) and IMiDs in the 2015–2020 period (37.6%).

As shown in Table 1, in each period, 20, 105, and 95 patients received ASCT as first-line therapy (24.7, 47.5, and 29.8%, *p* < 0.001). Among them, 13, 74, and 58 patients received upfront ASCT without any induction therapy (65.0, 70.5, and 61.1%, *p* = 0.372). For those who had a previous induction therapy, six patients in the pre-2010 period received melphalan-based induction therapy before ASCT, while 24 patients in 2010–2014 and 28 patients in 2015–2019 received IMiD-based chemotherapy before ASCT. In the patients who did not undergo ASCT, LDex (lenalidomide with dexamethasone) was the most extensively used regimen (48.0%). A proteasome inhibitor-based regimen, which was rarely used in POEMS syndrome before 2015, became one of the most important regimens for POEMS syndrome in 2015–2019. In 2015–2019, 24.1% of the patients used bortezomib (or ixazomib)-based therapy as the first-line treatment. The trends of the first-line treatment option in each period are shown in Fig. 1d and Supplementary figure 1.

**Fig. 1:**
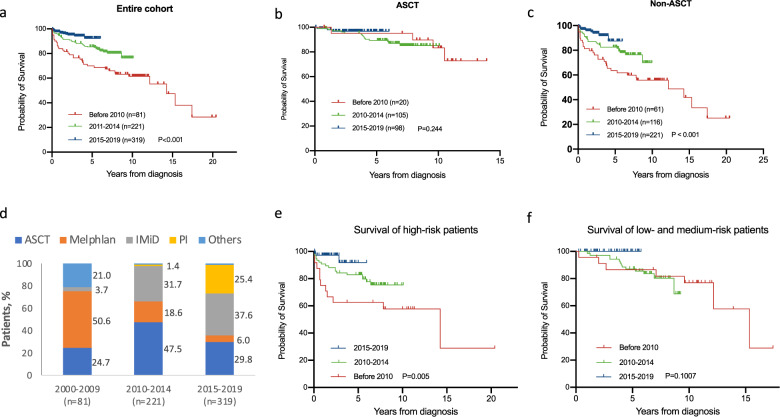
Changes in the treatment and outcomes in the past three periods. **a**. Kaplan–Meier curve for overall survival of entire cohort. **b** Kaplan–Meier curve for overall survival of patients who underwent ASCT by time period. **c** Kaplan–Meier curve for overall survival of patients who received a non-ASCT regimen by time period. **d** First-line treatment in all patients. **e** Kaplan–Meier curve for overall survival of high-risk patients. **f** Kaplan–Meier curve for overall survival of low- and medium-risk patients.

Within a median follow-up of 45 months (range, 1–245 months; 99, 75, and 48 months in each period), a total of 91 patients died, of which 34 were diagnosed before 2010, 41 were diagnosed during 2010–2014 and 16 were diagnosed during 2015–2019. The causes included disease progression-related renal failure (*n* = 11), cardiopulmonary failure (*n* = 13), disease progression not otherwise specified (*n* = 42), infection (*n* = 10), cachexia (*n* = 2), and others (*n* = 13).

The 5-year OS rates in the three periods were 70.0, 85.4, and 92.2% (95% CI, 58.6–78.8, 80.3–89.8, and 86.3–96.1; *p* < 0.05). The median overall survival from diagnosis for the entire cohort was 184 months (95% CI, 140–228 months), the median survival in the first group was 171 months (95% CI, 128–213 months), and the median OS in the latter two cohorts was not reached (Fig. 1a).

Among patients who received non-ASCT treatment, significant improvement of the overall survival was observed, with 5-year OS rates of 62.6% and 81.8% in the two former periods and a 5-year OS rate of 87.5% in the most recent period (Fig. 1c). The survival gap between ASCT and non-ASCT treatment showed a narrowing trend. In both the period before 2010 and the period of 2010–2014, people who underwent ASCT had longer overall survival than those who did not (Supplementary Figure 2; before 2010 median OS: undefined vs. 146 m, *p* = 0.036; 2010–2014 median OS: both undefined, *p* = 0.046). In 2015–2019, people who underwent ASCT had no significant difference in overall survival compared with those who did not (*p* = 0.126). Of note, in high-risk patients, the 5-year OS in each period was 60.0, 82.9, and 91.8% (*p* = 0.005). In contrast, among low- and medium-risk patients, the 5-year OS did not change significantly (*p* = 0.101) (Fig. 1e–f).

This study with a total sample size of 621 patients outlined the advances in the management of POEMS syndrome in the past two decades at our center. Misdiagnosis used to be very common in POEMS syndrome [10]. At our center, glucocorticoids were provided to 31.9% of patients before the diagnosis of POEMS syndrome was established. However, timelier diagnoses and proper treatments were observed over time, and more patients are diagnosed at an early stage with a lower disease burden. Moreover, this study also showed the dramatic changes in the treatment of POEMS syndrome in a real-world setting. In our practice, MDex used to be the most frequently used first-line therapy (50.6%) but was replaced by ASCT in 2011–2014 (47.5%) and then LDex in 2015–2019 (37.6%). Overall, the 5-year OS increased from 70.0 to 92.2%. The inflection point seems to have occurred around 2010. One of the reasons for the improvement is that more patients received ASCT as the first-line therapy (24.7, 47.5, and 29.8%; *p* < 0.001), which was also seen in the study describing the Mayo Clinic experience, in which 8 and 49% of the patients received ASCT before and after 2003, respectively [1]. The safety and efficacy of ASCT have been demonstrated in many studies in the past decade, especially after proper induction therapy [6, 11, 12]. In the study conducted by Zhao et al. [12], ASCT had the highest response and PFS rates compared with MDex and LDex. Of note, in high-risk patients, ASCT had a higher response rate than MDex and better PFS than LDex in medium- to high-risk patients. This also explains the improved survival of high-risk patients in this study. Moreover, in a study conducted by a Japanese group [6], 95% of the patients received more than one prior induction therapy. With a median follow-up of 72 months, the 5-year OS was 90.1%, and the 5-year PFS was 63.2%, which is comparable to the 5-year OS of 92.2% in this study. All of the above findings confirm that ASCT is still one of the best first-line treatments for POEMS syndrome. The other reason is the advent of novel agents, which greatly improved the survival for patients who did not undergo ASCT. The most profound change occurred with the replacement of melphalan by IMiDs after 2010. MDex has been used to treat POEMS syndrome for a long time with promising outcomes. [12] However, because of concerns about secondary malignancy and the suppression of hematopoiesis, MDex is not the first choice for young patients. In contrast, LDex can induce rapid hematological and neurological responses [7] and lead to a similar OS to ASCT and MDex [12], thus becoming the preferred option. In a prospective phase II study of low-dose LDex, it was suggested that patients with organ dysfunction may benefit more from lenalidomide because of its rapid function [7]. This regimen is also increasingly used in induction therapy before ASCT. Furthermore, a bortezomib-based regimen is also suggested to be a safe and effective regimen. He et al reported 20 patients who received a bortezomib-based regimen as first-line therapy, and none of them died or developed PD during a median follow-up of 11 months [13]. Although concerns about neurological side effects hamper the use of BDex, the favorable safety profile of this regimen shown in different studies verified its practical value, especially in patients with reduced renal function [14]. Overall, the efficacy gap between ASCT and non-ASCT regimens is reducing [12, 15]. Whether the same efficacy of ASCT can be achieved by non-ASCT regimens is yet to be determined, and more studies and longer follow-ups are needed to determine the results.

This study has limitations. The median follow-up in the 2015–2019 group was only 2 years, and the difference between the ASCT and non-ASCT groups may not be significant. In addition, as the response and relapse criteria were not standardized, the response rate and PFS were not analyzed in this study.

In conclusion, this study provides a thorough view of the changes in the management of POEMS syndrome in the past two decades. It is encouraging to see reduced misdiagnoses and earlier diagnoses. Overall survival increased significantly, mainly due to dramatic advances in non-ASCT therapeutic options. The increasing availability of drugs targeting plasma cells has changed the management of POEMS syndrome, and it is believed that more advances will be made in the future.

## Supplementary information

Treatment and outcomes of POEMS syndrome: Changes in the past 20 years
