# Supplementary material for: Treatment and outcomes of POEMS syndrome: changes in the past 20 years
Source: Blood Cancer J. 2021 Aug 14;11(8):145. doi: 10.1038/s41408-021-00540-1 (PMC8364551; doi:10.1038/s41408-021-00540-1)
Supplement: Supplementary file 1 — Treatment and outcomes of POEMS syndrome: Changes in the past 20 years [file 41408_2021_540_MOESM1_ESM.pdf]

Supplement

Supplemental Methods

Study design and patients

Patients who satisfied the POEMS syndrome diagnostic criteria described by Dispenzieri(1): 1) monoclonal plasma cell proliferative disorder and polyneuropathy as mandatory criteria; 2) one of the other major criteria (Castleman disease, sclerotic bone disease, and VEGF elevation); and 3) one of the minor criteria (organomegaly, extravascular volume overload, endocrinopathy, skin manifestations, papilledema, and thrombocytosis or thrombocytosis). Risk factors included age > 50 years, pulmonary hypertension, pleural effusion (each 1 point) and estimated glomerular filtration rate <30 ml/min/1.73 m2 (2 points). A total score of 0 is considered low risk, 1 is considered medium risk, and 2-4 is considered high risk. (2)

Statistical analyses

Overall survival (OS) was calculated from the time of diagnosis to death or the last follow-up. Baseline characteristics are presented as percentages and interquartile ranges (IQRs) for continuous data. Statistical analysis was performed with SPSS (version 26; IBM Corporation, NY). The chi-square test, or Fisher's exact test when appropriate, was used to determine the significance of differences in the values of categorical variables. Survival analysis was performed using the Kaplan-Meier method, and differences were compared with the log-rank test. *P*-values < 0.05 were considered significant.

Data sharing statement

For original data, please contact lijian@pumch.cn.

Supplementary Figure1. Induction among ASCT patients by time period.

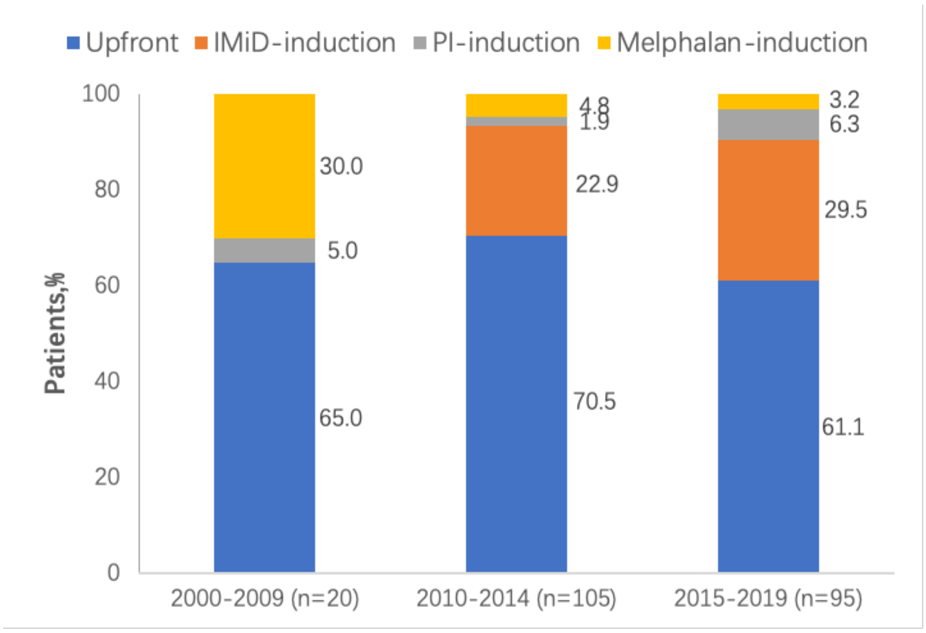

Supplementary figure 2. Overall survival of patients diagnosed in a. pre-2010, b. 2010-2014 and c. 2015-2019.

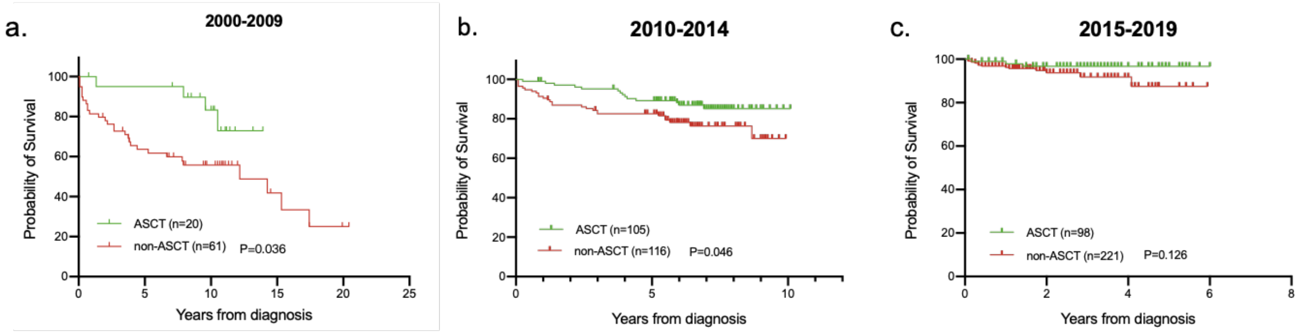

Reference

1. Dispenzieri A. POEMS syndrome: update on diagnosis, risk-stratification, and management. *American Journal of Hematology*. 2012;87(8):805-814.

2. Wang C, et al. Prognostic study for overall survival in patients with newly diagnosed POEMS syndrome. *Leukemia*. 2017;31(1):100-106.
